# A Modern Cohort of Duodenal Obstruction Patients: Predictors of Delayed Transition to Full Enteral Nutrition

**DOI:** 10.1155/2014/850820

**Published:** 2014-08-14

**Authors:** Sigrid Bairdain, David C. Yu, Chueh Lien, Faraz Ali Khan, Bhavana Pathak, Matthew J. Grabowski, David Zurakowski, Bradley C. Linden

**Affiliations:** ^1^Department of Pediatric Surgery, Boston Children's Hospital, Harvard Medical School, 300 Longwood Avenue, Fegan Building, 3rd Floor, Boston, MA 02215, USA; ^2^Department of Pediatric Surgery, Children's Hospital of Alabama, Birmingham, AL 35233, USA; ^3^Department of Medicine, Johns Hopkins University, Baltimore, MD 21218, USA; ^4^Department of Anesthesia, Boston Children's Hospital, Harvard Medical School, Boston, MA 02215, USA; ^5^Pediatric Surgical Associates, Children's Hospitals and Clinics of Minnesota, MN 55404, USA

## Abstract

*Background*. A common site for neonatal intestinal obstruction is the duodenum. Delayed establishment of enteral nutritional autonomy continues to challenge surgeons and, since early institution of nutritional support is critical in postoperative newborns, identification of patients likely to require alternative nutritional support may improve their outcomes. Therefore, we aimed to investigate risk factors leading to delayed establishment of full enteral nutrition in these patients. *Methods*. 87 patients who were surgically treated for intrinsic duodenal obstructions from 1998 to 2012 were reviewed. Variables were tested as potential risk factors. Median time to full enteral nutrition was estimated using the Kaplan-Meier method. Independent risk factors of delayed transition were identified using the multivariate Cox proportional hazards regression model. *Results*. Median time to transition to full enteral nutrition was 12 days (interquartile range: 9–17 days). Multivariate Cox analysis identified three significant risk factors for delayed enteral nutrition: gestational age (GA) ≤ 35 weeks (*P* < .001), congenital heart disease (CHD) (*P* = .02), and malrotation (*P* = .03). *Conclusions*. CHD and Prematurity are most commonly associated with delayed transition to full enteral nutrition. Thus, in these patients, supportive nutrition should strongly be considered pending enteral nutritional autonomy.

## 1. Introduction

The duodenum is a common location for intestinal obstruction in the newborn requiring surgical intervention [1]. Intrinsic obstruction ranges from complete atresia with a gap to membranous web and to stenosis. In contrast to small intestinal atresia, ontogeny is thought to be secondary to failure of luminal recanalization [2]. Treatment often consists of operative duodenoplasty or bypass with a duodenoduodenostomy and has traditionally been performed through a laparotomy; however, laparoscopic approach has previously been shown to be a viable option [3]. Overall mortality for this congenital anomaly has improved; moreover, associated medical conditions are now responsible for most deaths [4].

Prolonged postoperative enteral feeding intolerance continues to challenge surgeons caring for this cohort of patients. Since early institution of nutritional support is critical in postoperative newborns, identification of patients likely to require alternative nutritional support is likely of benefit to this subset of patients. Therefore, our aim was to describe the short term nutritional outcomes and investigate risk factors leading to delayed establishment of full enteral nutrition in a modern cohort of patients with duodenal atresia at a single-institution.

## 2. Materials and Methods

Following approval from our Institutional Review Board (IRB number M08-08-0381), we performed a retrospective review of records from 1998 to 2012 utilizing ICD-9 code for small intestine atresia (751.1) at Boston Children's Hospital (BCH). Patients were included if they had a primary surgical repair or surgical treatment for a complication of a previous repair of a newborn intrinsic duodenal obstruction during this 14-year period. We reviewed the following variables: gender, gestational age (GA), birth weight, Apgar 1-minute score, Apgar 5-minute score, and radiographic findings such as proximal dilatation, duodenal anatomy, technique of primary surgical repair, malrotation, annular pancreas, congenital heart disease (CHD), Down's syndrome, and esophageal atresia with or without tracheoesophageal fistula (EA/TEF).

Kaplan-Meier product-limit analysis with Greenwood estimators was used to calculate median time to full enteral nutrition, with the log-rank test to compare area under the survival curves [5]. Covariates were evaluated by univariate and multivariate analysis using the Cox proportional-hazards model to identify independent predictors with the likelihood ratio test to assess significance [6] and log-minus log plot to verify the assumption of proportional hazards over time [7]. Hazard ratios (HR) and 95% confidence intervals (CI) were determined for significant multivariate predictors of time to full enteral nutrition [8]. Statistical analysis was performed using IBM SPSS statistics (version 21.0, IBM, Armonk, NY). Two-tailed *P* < .05 was considered statistically significant. Power analysis indicated that the sample sizes of patients with potential risk factors provided 80% power for detecting a significant hazard ratio of 0.75 in delayed transition to full enteral nutrition (version 7.0, nQuery Advisor, Statistical Solutions, Saugus, MA).

## 3. Results

### 3.1. Demographics

A total of 87 patients were found to have an intrinsic duodenal obstruction and met inclusion criteria. Median age was 37 weeks (interquartile range [IQR]: 35–39 weeks). Median birth weight was 2680 grams (IQR: 2145–3200 grams). Median Apgar scores were 8 at 1 and 5 minutes (IQR: 7-8 and 8-9, resp.). There was a slight female predominance in the cohort (52% versus 48%). Proximal bowel dilatation was documented in 19% (16/86) of the patients (Table 1). A majority of the duodenal anatomy encountered was atresia (63%), followed by webs (24%) and stenosis (13%). Concurrent congenital anomalies included CHD (55%) and Down syndrome (38%) (Table 1). CHD was further subdivided into the following types encountered: 9 (19%) PDA; 21 (44%) ASDs and/or VSDs; 7 (15%) tetralogy of Fallot (TOF); 2 (4%) Coarctation of the aorta; 6 (12%) hypoplastic hearts or pulmonary arteries; 2 (4%) complete AV canal (CAVC); and 1 (2%) bicuspid aortic valves.

### 3.2. Primary Operative Characteristics

Choice of operation, as well as whether it was performed open or laparoscopically, was dictated by the choice of the operating surgeon. Five (6% or 5/86) of the operations were performed laparoscopically, while the remaining 81 were performed through a laparotomy. The techniques described for the primary duodenal repair included 58 duodenoduodenostomies, 6 duodenojejunostomies, 1 gastroduodenostomy, and 15 duodenoplasty repairs (with or without web excision), and 6 patients had web excision without duodenoplasty. One patient was not repaired given that she died two days into her hospital course from complex cardiac complications.

### 3.3. Intraoperative Findings: Single versus Multiple Intestinal Obstructions

Six (7% or 6/86) patients were found to have a second, distal, concurrent, and intestinal obstruction at the time of their proximal duodenal obstruction repair. Fifty-six percent of the operating surgeons performed a maneuver to detect a concurrent obstruction, while no maneuver was noted in the remaining 44% of operative notes. These maneuvers included inspection alone (30%), passage of small transanastomotic catheter (22%), and passage of transanastomotic catheter with small fluid bolus (3%). As a result of these maneuvers, the following anomalies were identified; two patients had a distal duodenal web, two had a second duodenal atresia, one had a jejunal atresia, and one had multiple distal atresias in both the duodenum and the jejunum.

### 3.4. Nutritional Perioperative Characteristics

Eighty patients received parenteral nutrition (PN). Seven patients did not receive PN; six patients never received PN and one patient never had the opportunity to receive PN. The cohort that received PN required a median of 10 days on PN (IQR: 6–12 days). The median time to 100% EN was 12 days (IQR: 9–17 days), excluding the one patient who died 2 days into her hospital course. The median time to 100% EN in those patients who never received PN was 7 days (range 5–26 days). 71 out of the 84 patients were transitioned to 100% oral feeds prior to discharge at a median of 13 days (IQR: 10–21 days (Table 2).

Thirteen patients received a transanastomotic feeding tube during their operation. Their median time on PN was 9 days (IQR: 5–16 days), with a full range 0–65 days, versus the larger cohort (*P* = .29, Mann-Whitney *U*-test). Regarding time to 100% enteral nutrition, the median time for those with feeding tubes was 9 days (IQR: 7–22 d), with full range 2–63 days, versus the larger cohort (*P* = .28, Mann-Whitney *U*-test). There were no anastomotic leaks reported in this smaller cohort of patients.

### 3.5. Univariate Analysis

Variables were tested as potential risk factors for delayed enteral feeding. Those included gender, GA ≤ 35 weeks, birth weight, Apgar 1 scores, duodenal anatomy, proximal bowel dilatation, technique of primary surgical repair, presence of feeding tube, malrotation, annular pancreas, CHD, Down's syndrome, and EA/TEF (Table 3). Of these, GA ≤ 35 weeks, birth weight, atresia anatomy, surgical repair, malrotation, and CHD were found to be significantly associated with delayed progression to full enteral feeding on univariate analysis.

### 3.6. Multivariate Analysis

Multivariate Cox regression analysis revealed three independent predictors of delayed progression to full EN. Those variables were gestational age 35 weeks or less (*P* < .001), CHD (*P* = .02), and malrotation (*P* = .03). Variables such as birth weight, atresia anatomy, and surgical repair were not found to be independent predictors of delayed progression to full enteral feeding. Patients were then further classified based on the presence or absence of CHD and then stratified by GA.

**Figure 1:**
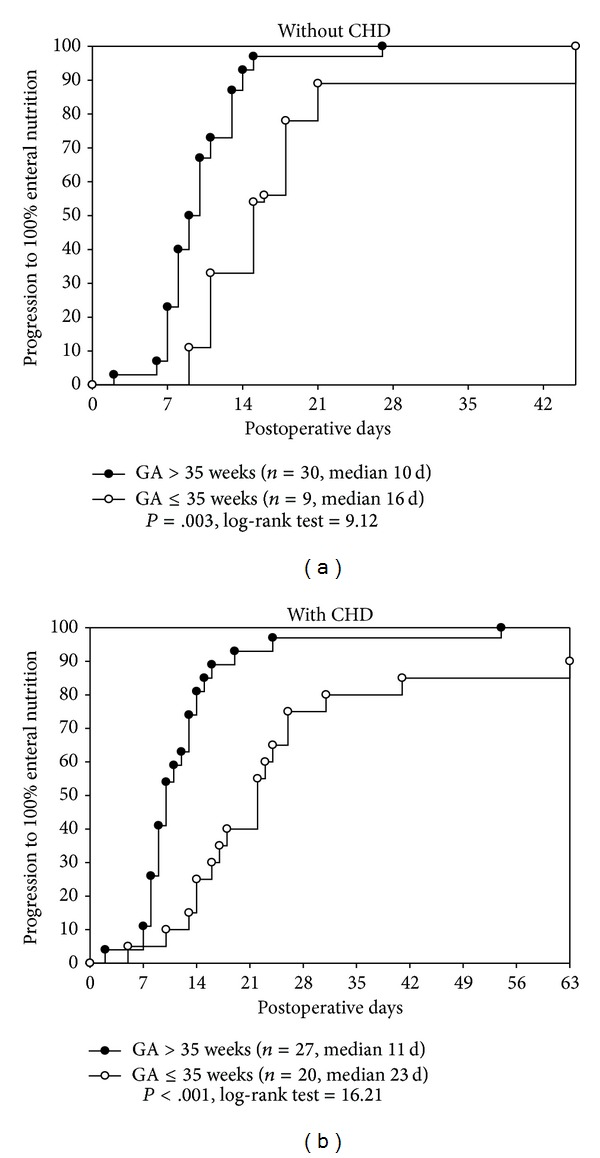
(a) Kaplan-Meier curves illustrating the progression to 100% enteral nutrition (100% EN) in patients without congenital heart disease (CHD). The importance of low gestational age (GA) is that if a patient was GA > 35 weeks (*n* = 30) then the median time to progression was 10 days (IQR: 8–12 days). However, if the GA ≤ 35 weeks (*n* = 9) then the median time to progression was 16 days (IQR: 11–20 days) (*P* = .003, log-rank test = 9.12). (b) Kaplan-Meier curves illustrating the progression to 100% enteral nutrition (EN) in patients with congenital heart disease (CHD) and illustrates the synergistic effect of CHD on prematurity. For those patients with GA > 35 weeks (*n* = 27) and CHD, the median time to progression was 11 days (IQR: 8–14 days). For those patients with GA ≤ 35 weeks (*n* = 20) and CHD, their median time to progression was 23 days (IQR: 15–30 days) (*P* < .001, log-rank test = 16.21). Both prematurity and the presence of CHD translated into a much longer delay in progression to 100% EN.

Among patients without CHD who had GA > 35 weeks (*n* = 30) the median time to 100% EN progression was 10 days (IQR: 8–12 days), whereas if GA ≤ 35 weeks (*n* = 9) the median time to progression was 16 days (IQR: 11–20 days) (*P* = .003, log-rank test = 9.12) (Figure 1(a)). Among patients with CHD who had GA > 35 weeks (*n* = 27), the median time to progression was 11 days (IQR: 8–14 days), whereas for those with GA ≤ 35 weeks (*n* = 20) the median time to progression was 23 days (IQR: 15–30 days) (*P* < .001, log-rank test = 16.21) (Figure 1(b)).

### 3.7. Short-Term Mortality and Outcomes

Three patients died, all of whom had significant CHD. The first patient died two days into her hospital course. The second patient, born with a hypoplastic left heart and aortic coarctation, died of uncontrollable pulmonary hypertension shortly following an open duodenal atresia repair. The third patient, born with tetralogy of Fallot (TOF) and a chromosome 12 deletion, died almost one year following surgery while being in the hospital for a cardiac procedure. Among the 84 survivors, the median followup was 20 months (IQR: 5–48 months).

There were 13 (15%) major surgical complications following initial operative experience (Table 4). The 13 surgical complications included six bowel obstructions, four anastomotic leaks, two late-strictures, and one incisional hernia. Twenty-four percent (10/42 patients) occurred between 1998 and 2005 versus 3% (3/44) between 2006 and 2012 (*P* = .04). The earlier cohort excluded one patient from analysis as she was an early mortality and was never repaired. None of the patients with anastomotic leaks had a feeding tube present.

Medical complications were also reviewed in this cohort. The medical complications included 2 episodes of line sepsis and one episode of necrotizing enterocolitis (NEC). Treatment of the medical complications was as follows: (1) the two episodes of line sepsis were treated with antibiotics; and (2) the one episode of NEC was treated with a 14-day course of antibiotics and bowel rest.

## 4. Discussion

This study describes a cohort of 87 patients who were treated for intrinsic duodenal obstruction in the newborn period at a single institution. This may be one of the largest series of repairs as compared to other previously published series [1]. Six patients (7%) did have a concurrent second intestinal obstruction, while 13 patients had intraoperative nasojejunal transanastomotic feeding tubes placed without anastomotic complications. Variables that had previously been associated with delayed transition to full enteral feedings were not important in our study, including proximal bowel dilatation, duodenal anatomy, or technique of primary surgical repair. We concluded that the independent predictors for longer postoperative progression to full enteral nutrition were prematurity, CHD, and malrotation in our cohort.

In this cohort of patients, the mortality rate across 14 years was 3%, which is comparable to the approximately 4% early operative mortality reported in the available literature [1, 2]. Presence of complex cardiac conditions, Down's syndrome, and other intestinal atresia and trachea-esophageal fistula has been reported to place these patients at higher risk of late morbidity and mortality [9]. 55% of patients in this cohort had CHD, while 38% patients had Down's syndrome. Contrary to the findings of worse outcomes with associated Down's syndrome, no such association was elucidated in this cohort [10].

Recent literature has suggested that the repair of duodenal atresia can be effectively and safely performed laparoscopically [11–15]. Our study only had 5 laparoscopic operations performed; however, many laparoscopic-based studies are suggesting an early return of bowel function congruent with laparoscopic procedures in general. For example, Spilde et al. showed that the length of postoperative hospitalization (20.1 versus 12.9 days; *P* = .01), time to initial feeding (11.3 versus 5.4 days; *P* = .002), and time to full oral intake (16.9 versus 9 days; *P* = .007) were shorter in the group undergoing laparoscopic repair versus open repair [11]. Further studies are needed to elucidate this point in particular.

As reported in this study, slightly prolonged transition time to full enteral feeding may be in part due to a greater proportion of patients undergoing open operative approach versus an inherent component of the study cohort, including the initiation of PN in 80 (92%) patients within the cohort. Similar to a recently published study, the small subset of patients (*n* = 6) in our study who never received PN had a faster transition to full enteral nutrition, 7 days versus 12 days for the entire cohort [16]. The two variables though, that had the greatest influence overall, were prematurity and presence of CHD; both were noted to be associated with increased odds of delayed transition to full enteral nutrition and outweighed all other variables. Even with this being a retrospective study and there being an absence of a standardized approach to feeding advancement, we do believe that there is the potential of significant benefit of early institution of appropriate nutritional support and using these risk factors to tailor the appropriate nutritional regimen.

From a surgical perspective, several important findings also arose from this study. First, intraoperatively, six patients (7%) were noted to have a second, distal, concurrent, and intestinal obstruction, representing a higher rate of concurrent intestinal obstruction as compared to (1–3%) the rate reported in the available literature [2, 13]. This does suggest that the evaluation for presence of concurrent obstruction may be worthwhile to avoid the morbidity associated with a missed lesion. Other major complications did include line sepsis in two patients; however, there was not an increased rate in the highest risk groups [10, 16]. Secondly, there was a small degree of a time-epoch phenomena. For example, it appeared that between the two epochs 1998–2005 and 2006–2012, there was a significant decrease in the number of associated in-hospital complications (*P* = .04) in the time period between 2006 and 2012. This may be due in part to both a learning curve and a higher volume of cases performed, which is congruent with more recent studies but further studies are needed [17].

Lastly, none of the patients in our cohort who had received a transanastomotic tube developed a leak or developed a stricture and had a slightly decreased transition to full enteral feeds. More recent studies have shown that use of a transanastomotic feeding tube decreases not only the postoperative time to initiation of enteral nutrition, but also the time to full enteral nutrition delivered proximal to the anastomosis [18–21]. There was a trend in decreased number of days within our subset of patients who received transanastomotic feeding tubes but did not remain on multivariate analysis. We do believe that with a larger number of patients and a standardized placement of these transanastomotic feeding tubes, we would anticipate a significant difference in transition to full enteral nutrition. Further studies on a larger scale are needed.

This study has several limitations. These include its respective design, despite medical records being reviewed in full detail. Another limitation was that there was no overt standardization for repair guidelines (open versus laparoscopic or type of repair employed), or whether a patient received a transanastomotic feeding tube versus an access line for nutrition. However, these patients were followed postoperatively with minimal loss to follow-up and no significant long-term complications.

## 5. Conclusion

Based on our findings, prematurity, CHD, and malrotation predict a longer time to reach full enteral nutrition; therefore, supportive nutrition is suggested. Furthermore, we did not see an associated increase in leak rate in the small subset of patients who received transanastomotic feeding tubes. Given the aforementioned findings and limitations, further studies are needed to elucidate a standardized methodology for nutritional support during clinical management of these patients in the setting of single or multiple intestinal obstructions. In the absence of prospectively collected data, these risk factors can be used to potentially identify patients with a greater risk of delayed transition to full enteral nutrition and therefore tailor their nutritional support regimen accordingly.

## Figures and Tables

**Table 1:** Patients' characteristics with duodenal obstruction (*N* = 87).

Characteristic	Median	IQR	Range
Age at surgery, d	5	2–12	0–730
Gestational age, weeks	37	35–39	25–42
Birth weight, grams	2680	2145–3200	737–7000
Apgar score 1-minute	8	7-8	0–9
Apgar score 5-minutes	8	8-9	5–10

	Number	Percentage	

Gender			
Female	45	52%	
Duodenal anatomy			
Web	21	24%	
Stenosis	11	13%	
Atresia	55	63%	
Proximal bowel dilation	16	19%	
Technique of primary surgical Repair			
Duodenoduodenostomies	58	68%	
Duodenojejunostomies	6	7%	
Gastroduodenostomy	1	1%	
Duodenoplasty	15	17%	
Web excision	6	7%	
Feeding tube	13	15%	
Intestinal malrotation	27	31%	
Annular pancreas	17	20%	
Congenital heart disease	48	55%	
Down's syndrome	33	38%	
Imperforate anus	3	3%	
Hirschsprung's disease	1	1%	
EA/TEF	5	6%	

EA/TEF = esophageal atresia/tracheoesophageal fistula. IQR = interquartile range.

**Table 2:** Nutritional characteristics of the cohort.

Characteristic	Median time	IQR	Range
Parenteral nutrition, d	10	6–12	0–258
Time to 100% EN, d	12	9–17	2–211
Time to 100% PO∗, d	13	10–21	3–69
Discharge time, d	17	12–31	0–1852

∗Based on the 71 who transitioned to 100% by mouth (PO). EN = enteral nutrition; IQR = interquartile range.

**Table 3:** Predictors of delayed transition to full enteral nutrition.

Variable	Univariate analysis	Multivariate Cox regression analysis		
	*P* value	Hazard ratio	95% CI	*P* value
Gender	.15			.18
GA ≤ 35 weeks	<.001	0.33	0.19–0.54	<.001∗
Birth weight	<.001			.79
Apgar, 1-min	.13			.52
Proximal dilatation∗∗	.60			.59
Duodenal anatomy	<.001			.32
Technique of primary surgical repair	<.01			.16
Feeding tube	.89			.76
Malrotation	.02	0.58	0.36–0.94	.03∗
Annular pancreas	.51			.47
Congenital heart disease	.003	0.59	0.37–0.93	<.02∗
Down's syndrome	.74			.71
EA/TEF	.14			.40

GA = gestational age, EA/TEF = esophageal atresia/tracheoesophageal fistula.

CI = confidence interval. ∗Statistically significant independent predictor of delayed transition. ∗∗Proximal bowel dilation noted on radiographic or intraoperative findings.

**Table 4:** Perioperative complications∗.

Complication	*n* (%)	Time period 1998–2005 (*N* = 42)	Time period 2006–2012 (*N* = 44)
Small bowel obstructions	6 (7%)	3	3
Anastomotic leaks	4 (5%)	4	0
Anastomotic stricture	2 (2%)	2	0
Incisional hernia	1 (1%)	1	0

Total	13 (15%)	10 (24%)	3 (7%)^†^

∗Based on a total of 86 patients (one patient was excluded due to early death at 2 days). ^†^Statistically significant lower complication rate since 2006 (*P* = 0.04, Fisher's exact test).
